# The Alteration of the Epidermal Basement Membrane Complex of Human Nevus Tissue and Keratinocyte Attachment after High Hydrostatic Pressurization

**DOI:** 10.1155/2016/1320909

**Published:** 2016-09-26

**Authors:** Naoki Morimoto, Chizuru Jinno, Atsushi Mahara, Michiharu Sakamoto, Natsuko Kakudo, Masukazu Inoie, Toshia Fujisato, Shigehiko Suzuki, Kenji Kusumoto, Tetsuji Yamaoka

**Affiliations:** ^1^Department of Plastic and Reconstructive Surgery, Kansai Medical University, Hirakata, Japan; ^2^Department of Plastic and Reconstructive Surgery, Graduate School of Medicine, Kyoto University, Kyoto, Japan; ^3^Department of Biomedical Engineering, National Cerebral and Cardiovascular Center Research Institute, Suita, Japan; ^4^Japan Tissue Engineering Co., Ltd., Gamagori, Japan; ^5^Department of Biomedical Engineering, Osaka Institute of Technology, Osaka, Japan

## Abstract

We previously reported that human nevus tissue was inactivated after high hydrostatic pressure (HHP) higher than 200 MPa and that human cultured epidermis (hCE) engrafted on the pressurized nevus at 200 MPa but not at 1000 MPa. In this study, we explore the changes to the epidermal basement membrane in detail and elucidate the cause of the difference in hCE engraftment. Nevus specimens of 8 mm in diameter were divided into five groups (control and 100, 200, 500, and 1000 MPa). Immediately after HHP, immunohistochemical staining was performed to detect the presence of laminin-332 and type VII collagen, and the specimens were observed by transmission electron microscopy (TEM). hCE was placed on the pressurized nevus specimens in the 200, 500, and 1000 MPa groups and implanted into the subcutis of nude mice; the specimens were harvested at 14 days after implantation. Then, human keratinocytes were seeded on the pressurized nevus and the attachment was evaluated. The immunohistochemical staining results revealed that the control and 100 MPa, 200 MPa, and 500 MPa groups were positive for type VII collagen and laminin-332 immediately after HHP. TEM showed that, in all of the groups, the lamina densa existed; however, anchoring fibrils were not clearly observed in the 500 or 1000 MPa groups. Although the hCE took in the 200 and 500 MPa groups, keratinocyte attachment was only confirmed in the 200 MPa group. This result indicates that HHP at 200 MPa is preferable for inactivating nevus tissue to allow its reuse for skin reconstruction in the clinical setting.

## 1. Introduction

High hydrostatic pressure (HHP) technology allows cells or tissues to be inactivated without the use of chemicals such as detergents. The technology has been used to produce various decellularized tissues [1–3]. The advantages of HHP treatment are that the processing time is short (within 10 min) and that the effects are uniform regardless of the thickness or hardness of tissue. We previously reported that HHP treatment for 10 min at pressures of higher than 200 MPa can completely inactivate human and porcine skin [4, 5]. The dermal collagen fibers of human skin, which was pressurized at up to 1000 MPa, showed no apparent damage under scanning electron microscopy (SEM), and the epidermal basement membrane could be detected by the immunohistochemical staining of type IV collagen [5, 6]. Thus, we tried to reconstruct skin using a combination of skin specimens which were subjected to pressures of higher than 200 MPa, which had no viable cells, including keratinocytes, and cultured epidermal autografts which had been used clinically in the treatment of patients with extended burns [7, 8]. Human cultured epidermis (hCE) engrafted and survived on human skin pressurized at 200 MPa but failed to take on the human skin pressurized at 1000 MPa [5].

Regarding skin reconstruction, the treatment of giant congenital melanocytic nevi (GCMN) remains a challenge in the field of plastic and reconstructive surgery [9]. GCMN of more than 20 cm in diameter are reported to occur in approximately one in 20,000 newborns and transform into malignant melanomas in 0.7% to 8.2% of cases [8–12]. Histologically, nevus cells are present in the entire layer of the dermis and, in some cases, the subcutaneous tissue. The removal of full thickness of the nevus tissue is therefore necessary to prevent the emergence of melanoma [12]. We previously reported that all of the cells in nevus tissue were inactivated after HHP at pressures of higher than 200 MPa, as well as in normal human skin, and that the hCE survived on the pressurized nevus after pressurization at 200 and 500 MPa but not at 1000 MPa. In tissue that was pressurized to 1000 MPa, the immunohistochemical staining of type IV collagen indicated the preservation of the basement membrane [6].

In the present study, we further explore the changes of the epidermal basement membrane of human nevus and elucidate the cause of the difference in hCE engraftment by the immunohistochemical staining of laminin-332 (formerly termed laminin-5) [13] which is the key component in epidermal attachment and type VII collagen (a main component of anchoring fibrils) and transmission electron microscopy (TEM) [14–16]. We then compared the attachment of human keratinocytes to the pressurized nevus after HHP at pressures of up to 1000 MPa.

## 2. Materials and Methods

Our protocol was approved by Kyoto University Graduate School and Faculty of Medicine Ethics Committee and the ethics committees of the National Cerebral and Cardiovascular Center Research Institute. For the human specimens, the HHP procedure was performed at the National Cerebral and Cardiovascular Center Research Institute. The animal experiments were performed at Kyoto University. Human nevus tissue and skin were taken from patients who underwent surgery at Kyoto University Hospital.

### 2.1. Preparation of Nevus Tissue

Nevus tissue specimens were obtained from three patients (mean age: 7.5 years; range: 7 months to 15 years) who underwent the surgical removal of nevi at Kyoto University Hospital. After removing the subcutaneous adipose tissues with scissors, full-thickness nevus tissue samples of 8 mm in diameter were prepared using an 8 mm biopsy punch (Kai Industries Co., Ltd., Gifu, Japan). The prepared specimens were preserved at 4°C in Dulbecco's modified Eagle's medium (DMEM; Life Technologies Japan, Ltd., Tokyo, Japan) until the pressurization process.

### 2.2. Pressurization of the Nevus Specimens by HHP

The 8 mm nevus specimens were divided into five groups based on the level of pressurization to which they were subjected: control, 100 MPa, 200 MPa, 500 MPa, and 1000 MPa. Specimens in the control group were removed from DMEM and preserved in a plastic bag filled with normal saline solution (NSS; Otsuka Pharmaceutical Co., Ltd., Tokyo, Japan) without pressurization at room temperature. The other specimens were packed in a plastic bag filled with NSS and each bag was immersed in transmission fluid in the chamber of a cold isostatic pressurization machine (Dr. CHEF, Kobe Steel, Ltd., Kobe, Japan) [1–6]. These specimens were pressurized at 100, 200, 500, or 1000 MPa for 10 min according to our previously reported procedure [1–6]. The pressure inside the chamber was increased at a rate of 65.3 MPa/min until it reached the target pressure. The target pressure was then maintained for 10 min and decreased to atmospheric pressure at the same rate.

### 2.3. Implantation of the Pressurized Nevus with hCE into the Subcutis of Nude Mice

Human cultured epidermis (hCE) was prepared by the Japan Tissue Engineering Co., Ltd. (J-TEC) using keratinocytes cultured from human neonatal foreskin using Green's method [17]. The epidermis of the pressurized nevus specimens in the 200, 500, and 1000 MPa groups was removed during the pressurization process; thus pressurized specimens from these groups were used in this experiment. A 1 × 1 cm square of hCE was placed on an 8 mm nevus specimen from each of the pressurized groups (*n* = 12; *n* = 4 in each group) and implanted into the subcutis on both sides in 7-week-old male BALB/c nude mice (Shimizu Laboratory Supply, Kyoto, Japan) (*n* = 6; two grafts per mouse), as we reported in our previous studies [5, 6]. Fourteen days after implantation, the mice were anesthetized with the inhalation of 2% isoflurane (Wako Pure Chemical Industries, Ltd., Osaka, Japan) and sacrificed by carbon dioxide inhalation and specimens were taken. The specimens were fixed with 10% formalin and embedded in paraffin blocks. Next, 5 mm paraffin sections were subjected to immunohistochemical staining for laminin-332 and type VII collagen.

### 2.4. The Immunohistochemical Staining of the Pressurized Nevus Specimens for Laminin-332 and Type VII Collagen

The specimens in control group and the pressurized groups immediately, both immediately after HHP and after implantation with hCE, were subjected to immunohistochemical staining for laminin-332 and type VII collagen. The unpressurized 8 mm specimens and the specimens immediately after pressurization were fixed with 10% neutral-buffered formalin solution and embedded in paraffin blocks. 5 mm thick sections were prepared from the central area of each sample. After deparaffinization and rehydration, antigen retrieval processing was performed using proteinase K (Dako Japan Co., Ltd., Tokyo, Japan) diluted threefold with Tris-Buffered Saline (TBS: Trizma® Pre-set crystals, Sigma-Aldrich Japan Co. Ltd., Tokyo, Japan) for 5 min at room temperature. The sections were rinsed twice in distilled water (DW; Life Technologies Japan, Ltd., Tokyo, Japan) and immersed in 3% hydrogen peroxide (Wako Pure Chemical Industries, Ltd., Osaka, Japan) for 10 min to block endogenous peroxidase activity. After the rinsing of the sections in DW and TBST [Tris-Buffered Saline (Sigma-Aldrich Japan Co. Ltd., Tokyo, Japan) with 0.05% Tween®20 (Polyoxyethylene Sorbitan Monolaurate, Nacalai Tesque, Inc., Kyoto, Japan) and 0.15 M NaCl (Sigma-Aldrich Japan Co. Ltd., Tokyo, Japan)], serum-free protein block (Code X0909, Dako Japan Co. Ltd., Tokyo, Japan) was applied for 5 min to block nonspecific protein binding.

The sections were incubated with mouse monoclonal anti-laminin-5 (laminin-332) antibodies (dilution 1 : 800; Abcam®, Abcam plc., Tokyo, Japan) as primary antibodies for 1 hour at room temperature. After rinsing in TBST, Simple Stain MAX-PO (MULTI) (Histofine®; Nichirei Bioscience, Inc., Tokyo, Japan) was applied for 30 min. The sections were rinsed again in TBST, exposed to DAB (3-3′-diaminobenzidine tetrahydrochloride; Nichirei Bioscience, Inc., Tokyo, Japan), and counterstained with hematoxylin. For the staining of anti-type VII collagen, mouse monoclonal anti-collagen VII antibodies (dilution 1 : 500; Abcam®, Abcam plc., Tokyo, Japan) were applied to the sections as primary antibodies for 2 h at room temperature. After rinsing in TBST, Simple Stain MAX-PO (MULTI) was applied for 30 min. The sections were rinsed again with TBST, exposed to DAB, and counterstained with hematoxylin.

Microphotographs were taken using a fluorescence microscope (Biorevo BZ-9000; Keyence, Co., Tokyo, Japan) at 200x magnification.

## 3. Transmission Electron Microscopy (TEM)

Tissue blocks, obtained from the nevus tissue, including its dermoepidermal junction, were fixed with 2.5% glutaraldehyde at 4°C overnight, postfixed with 1% osmium tetroxide (cacodylate buffer, pH 7.4) for 2 h at 4°C, dehydrated with ethanol, and embedded in Epon 812 resin. Ultrathin sections, which were cut perpendicular to the epidermal surface, were doubly stained with uranyl acetate and lead citrate and then observed by TEM (JEM-1400Plus, JEOL Ltd., Tokyo, Japan).

### 3.1. The attachment of Human Keratinocytes on Pressurized Nevus Specimens

A small piece of normal skin from a 22-year-old female patient that had been discarded after scar revision surgery at Kyoto University Hospital was used after obtaining written informed consent and keratinocytes were cultured using Green's method. The procedure was performed by Japan Tissue Engineering Co., Ltd. (J-TEC) [7, 17]. The keratinocytes that were used in this study had been passaged twice.

Nevus specimens of 4 mm in diameter (*n* = 16) were prepared using a 4 mm biopsy punch (Kai Industries Co., Ltd., Gifu, Japan) from the 8 mm nevus specimens of the four groups (control and 200, 500, and 1000 MPa). The nevus specimens were divided into four groups, control and 200, 500, and 1000 MPa groups (*n* = 4 in each group), and pressurized as described previously. The specimens were then placed into a cell-culture insert with an inner diameter of 6 mm and a transparent PET membrane with 0.4 *μ*m pores (Falcon, BD Biosciences, Bedford, USA). A cell suspension containing 1 × 10^4^ keratinocytes in 100 *μ*L of the keratinocyte basal medium with KGM-Gold with SingleQuots (KBM-Gold; Lonza Ltd., Basel, Switzerland) was added to the insert, and 500 *μ*L of the medium was also added after incubation for 3 h. The medium was changed after 3 days and the plates were incubated for 6 days at 37°C in a humidified atmosphere containing 95% air and 5% CO_2_. After cultivation, the tissues were washed with phosphate-buffered saline (PBS; Dako Japan Co., Ltd., Tokyo, Japan) and fixed with 3.7% formaldehyde solution for 10 min at room temperature. The cells were then immersed in 0.1% Triton X in phosphate buffer for 5 min. The treated cells were stained with rhodamine phalloidin (Life technologies, Grand Island, NY, USA) and DAPI solution (Dojin Chemical Co., Kumamoto, Japan). After staining, specimens were observed using a confocal laser scanning microscope (CLSM; FV1000-D, Olympus, Tokyo, Japan).

For the histological examination of the nevus tissue, the tissues were fixed with 10% formalin (Wako Pure Chemical Industries, Ltd., Osaka, Japan), and samples were embedded in paraffin. The paraffin-embedded tissues were then sectioned and stained with hematoxylin-eosin and subjected to immunohistochemical staining for laminin-332 and type VII collagen according to the above-stated methods.

## 4. Results

### 4.1. The Immunohistochemical Staining of Pressurized Nevus Specimens Immediately after HHP

**Figure 1:**
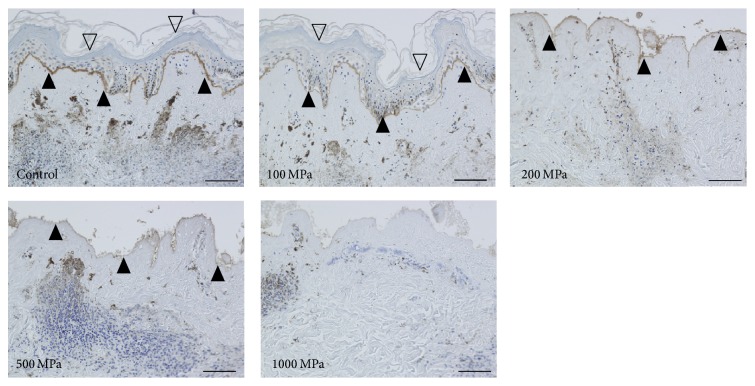
Micrographs of the immunohistochemical staining of type VII collagen. The open arrowheads indicate the remaining original epidermis of the nevus specimens in the control and 100 MPa groups. The closed arrowheads indicate the stained type VII collagen. The type VII collagen was clearly stained in the control, 100 MPa, and 200 MPa groups and weakly stained in the 500 MPa group but was not stained in 1000 MPa group. Scale bar = 100 *μ*m.

**Figure 2:**
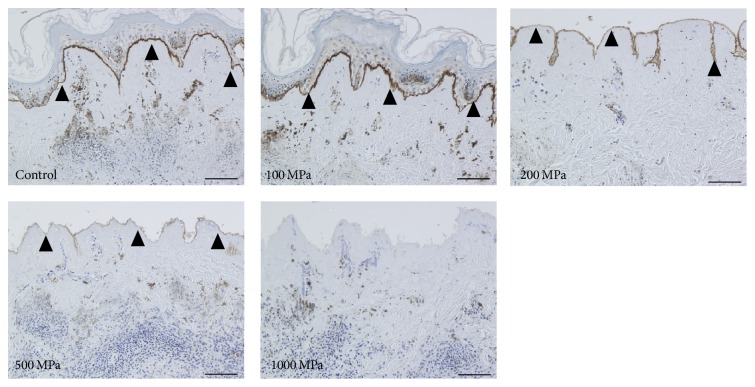
Micrographs of the immunohistochemical staining of laminin-332. The closed arrowheads indicate the stained laminin-332 in the basement membrane. Laminin-332 was clearly stained in the control, 100 MPa, and 200 MPa groups and weakly stained in the 500 MPa group but was not stained in the 1000 MPa group. Scale bar = 100 *μ*m.

The original epidermis of the nevus specimens remained intact in the control and 100 MPa groups. The epidermis was removed during the HHP process in the 200, 500, and 1000 MPa groups. The immunohistochemical staining of type VII collagen showed that the anchoring fibrils were clearly stained in the control and 100 and 200 MPa groups; weakened staining was observed in the 500 MPa group (Figure 1). In contrast, anchoring fibrils were not observed in 1000 MPa group (Figure 1; 1000 MPa). Laminin-332 was clearly confirmed by immunohistochemical staining in the control, 100 MPa, and 200 MPa groups, while the 500 MPa group was weakly stained (Figure 2). Laminin-332 was not observed in the 1000 MPa group (Figure 2; 1000 MPa).

### 4.2. Transmission Electron Microscopy (TEM) of the Dermoepidermal Junction

**Figure 3:**
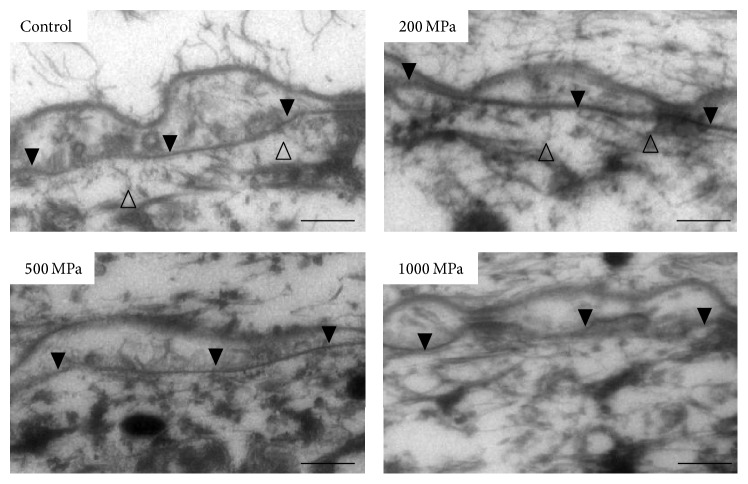
TEM micrographs of the dermoepidermal junction of the pressurized nevus. The closed arrowheads indicate the lamina densa in the basement membrane, which was observed in all specimens. The open arrowheads indicate the anchoring fibrils, which were observed in the control and 200 MPa groups but not in the 500 MPa or 1000 MPa groups. Scale bar = 2 *μ*m.

The lamina densa in the basement membrane was observed in all specimens (Figure 3). The lamina densa was found to mainly be composed of type IV collagen, which is consistent with our previous result, which confirmed the presence of type IV collagen in human skin and nevus specimens after HHP at pressures of up to 1000 MPa [5, 6]. In Figure 3, the open arrowheads indicate anchoring fibrils, which were observed in the control and 200 MPa groups but were not clearly observed in the 500 or 1000 MPa groups. This is consistent with the type VII collagen immunohistochemical staining results in the present study.

### 4.3. The Immunohistochemical Staining of the Pressurized Nevus Specimens with hCE after Implantation

**Figure 4:**
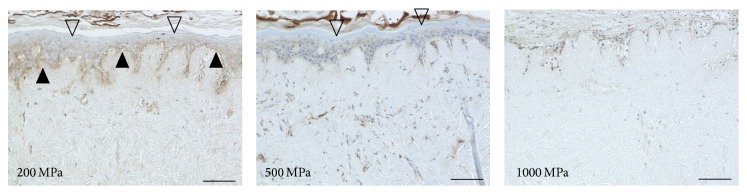
Micrographs of the immunohistochemical staining of type VII collagen in the nevus specimens with CE after implantation. The open arrowheads indicate the implanted CE in the 200 MPa and 500 MPa groups. The closed arrowheads indicate the stained type VII collagen. The type VII collagen was clearly stained in the 200 MPa group but not in the 500 MPa or 1000 MPa groups. Scale bar = 100 *μ*m.

**Figure 5:**
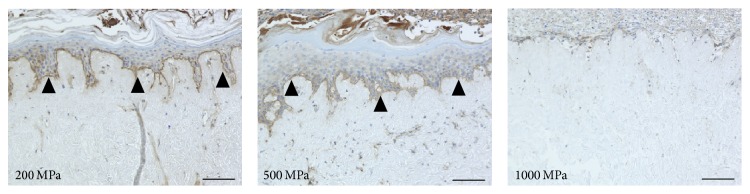
Micrographs of the immunohistochemical staining of laminin-332 in the nevus specimens with CE after implantation. The closed arrowheads indicate the stained laminin-332. Laminin-332 was clearly stained in the 200 MPa and 500 MPa groups but not in the 1000 MPa group. Scale bar = 100 *μ*m.

The hCE samples that were implanted on the pressurized nevus specimens survived only in the 200 MPa and 500 MPa groups (Figures 4 and 5). The positive staining of type VII collagen and laminin-332 was confirmed in the 200 MPa group. The staining of laminin-332 was observed in the 500 MPa group. Neither type VII collagen nor laminin-332 was stained in the 1000 MPa group.

### 4.4. The Attachment of Human Keratinocytes on the Pressurized Nevus Specimens

**Figure 6:**
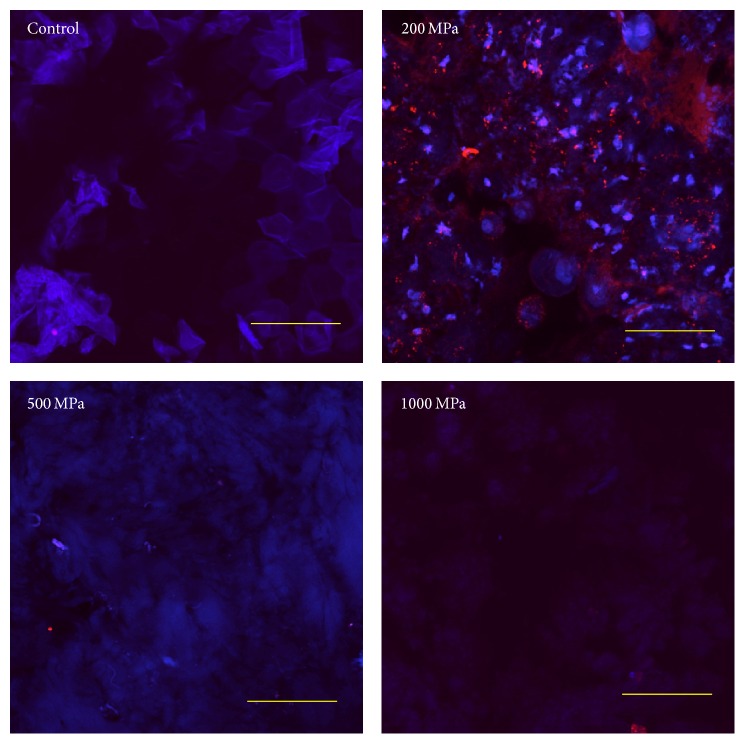
CLSM images of keratinocytes on the pressurized nevus specimens after 6 days of culturing. Actin fibers and nuclei were stained with rhodamine phalloidin and DAPI, respectively. Only the 200 MPa group was clearly stained. Scale bar = 50 *μ*m.

The CLSM images of human keratinocytes on the pressurized nevus specimens after 6 days of culturing only showed the clear staining of actin fibers and nuclei with rhodamine phalloidin and DAPI, respectively, in the 200 MPa group (Figure 6). This suggested that the seeded keratinocytes adhered to and spread out on the pressurized nevus of the 200 MPa group [18].

### 4.5. The HE and Immunohistochemical Staining of the Pressurized Nevus after Culturing

**Figure 7:**
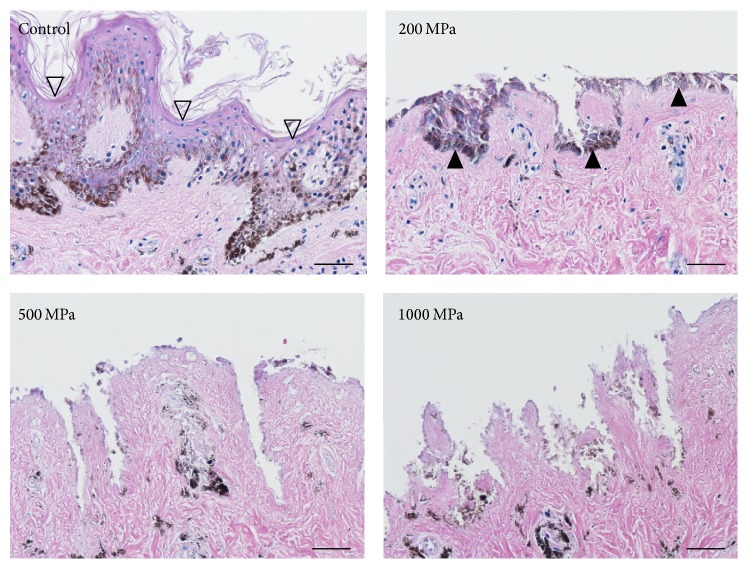
Micrographs of the HE-stained sections of the pressurized nevus specimens after 6 days of culturing. The original epidermis of the nevus specimens remained intact and seeded keratinocytes were not observed in the control group. The original epidermis was removed in the 200, 500, and 1000 MPa groups. The attachment of seeded keratinocytes on the pressurized nevus was only observed in the 200 MPa group. The open arrowheads indicate the original epidermis. The closed arrowheads indicate the seeded keratinocytes. Scale bar = 50 *μ*m.

**Figure 8:**
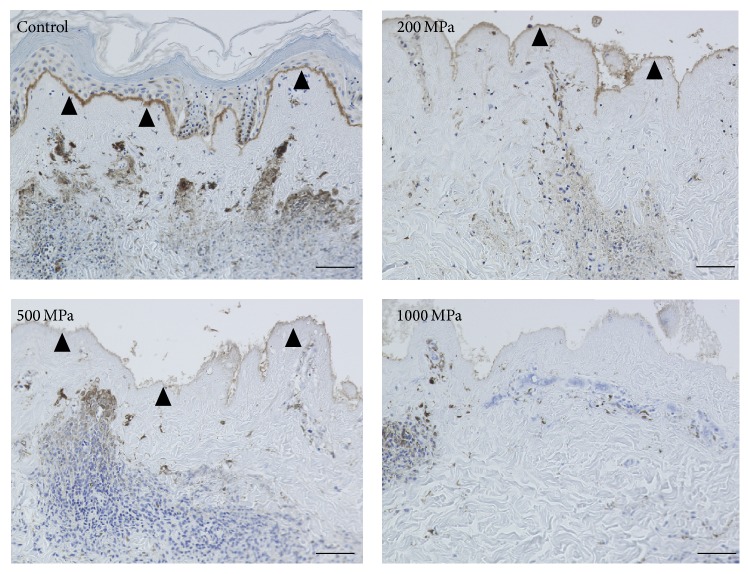
Micrographs of immunohistochemical staining of type VII collagen in the nevus specimens after 6 days of culturing. The closed arrowheads indicate the stained type VII collagen. The anchoring fibrils were clearly stained in the control and the 200 MPa groups and were weakly and locally stained in the 500 MPa group but were not stained in the 1000 MPa group. Scale bar = 50 *μ*m.

**Figure 9:**
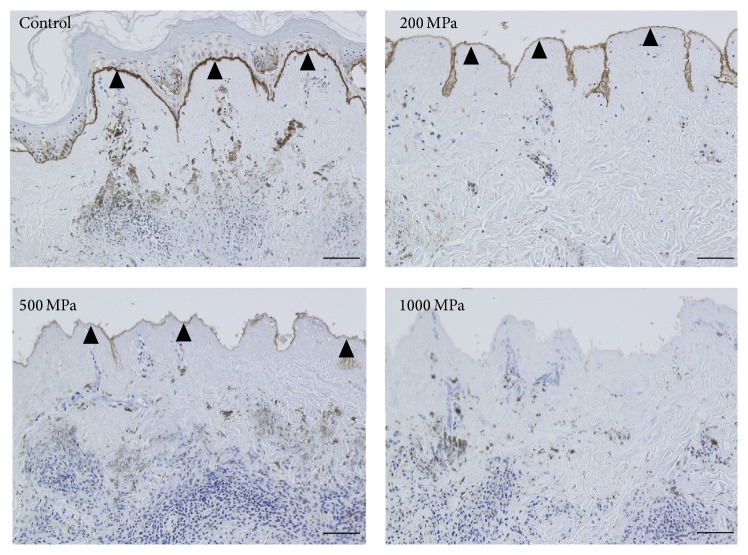
Micrographs of immunohistochemical staining of laminin-332 in the nevus specimens after 6 days of culturing. The closed arrowheads indicate the stained laminin-332. Laminin-332 was clearly stained in the control and 200 MPa groups and weakly and locally stained in the 500 MPa group but was almost completely unstained in the 1000 MPa group. Scale bar = 50 *μ*m.

The original epidermis of the nevus specimens remained intact and seeded keratinocytes were not observed in the control group (Figure 7; control). As indicated in the CLSM images, the seeded keratinocytes were only observed in the 200 MPa group (Figure 7; 200 MPa). Type VII collagen and laminin-332 were stained in the control, 200 MPa, and 500 MPa groups (Figures 8 and 9). Neither type VII collagen nor laminin-332 was positively stained in the 1000 MPa group (Figures 8 and 9).

## 5. Discussion

HHP at pressures of greater than 600 MPa has been reported to inactivate cells, tissues, and most pathogens. We previously reported that HHP at 1000 MPa could inactivate heart valves, blood vessels, cornea, and bone/bone marrow without damaging the native extracellular matrix [2, 9, 19–21]. We therefore attempted to inactivate skin and nevus tissue by HHP at 1000 MPa. In our previous study, we showed that that hCE survived on the pressurized nevus at 200 MPa and 500 MPa but not at 1000 MPa, despite the fact that the type IV collagen of the epidermal basement membrane remained intact [5, 6]. This suggested that HHP at 1000 MPa damaged another component of the basement membrane complex (other than type IV collagen) and inhibited the attachment of hCE. In this study, TEM of the basement membrane complex after HHP at pressures of up to 1000 MPa demonstrated that the remaining lamina densa was mainly composed of type IV. This finding was compatible with the results of our previous immunostaining experiments. On the other hand, laminin-332 was clearly stained in the basement membrane after HHP at pressures of up to 200 MPa and only weakly stained at 500 MPa. Lamina-332 staining was not observed at 1000 MPa. Similarly, type VII collagen was clearly confirmed after HHP at up to 200 MPa, both by immunostaining and by TEM. The presence of type VII collagen was not confirmed after HHP at pressures of more than 500 MPa. Laminin-332 is a major adhesion protein in the skin basement membrane and is crucial for the attachment of keratinocytes to the pressurized and inactivated nevus [22]. The laminin-332 staining results were well correlated with the results of hCE engraftment and survival on the pressurized nevus from our previous study [5, 6].

The attachment of keratinocytes was confirmed on the pressurized nevus after HHP at 200 MPa but not at 500 MPa. Interestingly, hCE engraftment was observed at 500 MPa. In our preliminary investigations, we grafted hCE on the nevi samples that were subjected to pressurization at various pressures* in vitro* and cultured them for 10 days; however, none of the samples attached or survived on the pressurized nevus. Thus, we seeded keratinocytes in the present study. However, our results showed that neither hCE samples nor keratinocytes easily survived* in vitro*. Our results suggest that HHP at 500 MPa has an adverse effect on the skin basement membrane and that HHP at 200 MPa is therefore preferable in clinical treatments to inactivate nevus tissue for reuse in skin reconstruction.

As for the stability of type IV collagen and laminin-332, collagen type IV molecules are covalently crosslinked by disulfide bridges via their C-terminal noncollagenous domain and N-terminal globular domain and are sufficiently stable to have high chemical resistance [23–25]. This stability is the reason why type IV collagen remained even after HHP at 1000 MPa, while the laminin-332 molecule, which is essential for the initial step of keratinocyte attachment, was damaged after HHP at pressures of greater than 500 MPa. Laminin-332 usually binds to type VII collagen in the basement membrane complex; thus, the disappearance of type VII collagen might also be involved in the insufficient attachment of hCE or keratinocytes after HHP at pressures of higher than 500 MPa.

## 6. Conclusion

The immunohistochemical staining and TEM results indicated that the type IV collagen of the basement membrane complex remained stable, even after HHP at 1000 MPa. In contrast, laminin-332 and type VII collagen were damaged after HHP at pressures of higher than 500 MPa. In the clinical setting, HHP at 200 MPa will be preferable for the inactivation of nevus tissue in order to allow its reuse in skin reconstruction.
